# Beyond visibility: gendered yield disparities in cowpea and soybean production in Nigeria

**DOI:** 10.3389/fsoc.2026.1833956

**Published:** 2026-10-29

**Authors:** Djana B. Mignouna, Lucky O. Omoigui, Oluyemi T. Akintayo, Beatrice A. Aighewi, Stephen Kyei-Boahen, Adebayo A. Akinola

**Affiliations:** 1 International Institute of Tropical Agriculture, Accra, Ghana; 2 International Institute of Tropical Agriculture, Kano, Nigeria; 3 École Supérieure D’Agronomie, Université de Lomé, Lomé, Togo; 4 International Institute of Tropical Agriculture, Abuja, Nigeria; 5 International Institute of Tropical Agriculture, Nampula, Mozambique; 6 Department of Agricultural Economics, Obafemi Awolowo University, Ile-Ife, Nigeria

**Keywords:** cowpea, decision-making, gender yield gap, Nigeria, resource endowments, soybean

## Abstract

**Introduction:**

Gender inequalities in agriculture may persist despite increasing recognition of women’s participation. However, evidence on the magnitude and observable sources of gender-associated yield gaps in Nigeria’s cowpea and soybean production systems remains limited. This study examines gender differences in resource access and agricultural decision-making, and estimates yield differentials between male- and female-headed farms.

**Methods:**

A cross-sectional survey was conducted between October and December 2022 across six major legume-producing states in Nigeria, involving 1,010 respondents. Farmer observations that met the required outcome and covariate criteria were used in the econometric analyses. Propensity Score Matching (PSM) and Oaxaca–Blinder decomposition were employed to estimate conditional gender-associated yield differences and assess the contribution of observed endowments.

**Results:**

PSM revealed a conditional male yield advantage of 429.34 kg/ha (SE = 253.01; *t* = 1.70; *p* < 0.10). Oaxaca-Blinder decomposition estimates mean yields of 730.44 kg/ha for female-headed farms and 1,070.80 kg/ha for male-headed farms, yielding a significant female–male differential of −340.37 kg/ha (*p* < 0.05). The endowment component (−438.91 kg/ha; *p* < 0.01), whereas the coefficient and interaction components were not statistically significant.

**Discussion:**

The findings suggest that differences in observed productive endowments contribute significantly to gender-associated yield disparities. However, they do not establish causal relationships between yield differences and inequalities in land rights, seed access, credit, extension services, or decision-making authority. The study highlights the importance of distinguishing women’s agricultural participation and visibility from their access to productive resources and effective control over agricultural decisions.

## 1 Introduction

Despite the importance of cowpea and soybean as a fundamental pillar for food security and rural livelihoods, factors beyond agronomic conditions influence productivity outcomes in these value chains (Omoigui et al., 2020; Kamara et al., 2022). Gender influences farmers’ productive opportunities through differences in access to and control over resources and participation in agricultural decision-making. Across sub-Saharan Africa, women farmers generally cultivate smaller landholdings and have more limited access than men to improved seed, fertilizer, credit, extension, mechanization, and market prospects. Such inequalities are embedded within household relations, social norms, land-tenure arrangements, inheritance practices, and agricultural institutions that influence who controls productive assets, makes production and commercialization decisions, and benefits from agricultural income (Olakojo, 2017). In this study, structural gender inequality refers to persistent differences in access, control, and decision-making opportunities that arise from gendered household relations and broader institutional arrangements rather than from individual choices alone. These may include intra-household bargaining processes, land tenure and inheritance institutions, gender norms, agricultural and market institutions, and access to services. Women’s empowerment and intra-household bargaining perspectives therefore, distinguish participation in agricultural production from effective control over productive resources and decisions. Women can play a significant role in farming, yet often have limited authority over land, production decisions, marketing, and the income generated by these activities. This situation does not just reflect the roles women choose within the household; rather, it highlights a form of participation that can be unequal or even exploitative, shaped by gendered power dynamics, household bargaining, and traditional systems of land tenure and inheritance (Mekuriaw, 2022). These structural factors create a vital sociological backdrop for understanding how gender affects productivity, even though the data we have does not allow us to fully quantify these mechanisms using econometric methods.

The distinction between participation, visibility, and empowerment is therefore important. Participation refers to women’s involvement in agricultural activities, while visibility concerns their recognition and observable presence within productive and decision-making domains (Ilesanmi, 2018). Empowerment goes beyond both concepts to women’s substantive capacity to exercise agency, influence decisions, control productive resources, and benefit from their participation. As a result, women can be very active and visible in agricultural production without having the corresponding authority to make decisions or control resources and income. In this study, visibility is used as a conceptual rather than a composite quantitative measure and is reflected descriptively through women’s representation and participation in land ownership, resource control, production and commercialization decisions, and control over income. Our analysis operates on two interconnected levels. First, we use survey indicators descriptively to capture the gendered landscape of production and decision-making. Second, the econometric analysis has a more focused aim: to estimate yield differences by gender, accounting for factors such as farm size, age, and education. These variables were chosen not only because they were consistently measured across our sample but also because they reflect important theoretical dimensions of productive endowments: farm size captures access to a fundamental agricultural asset and production scale; education reflects human capital that may influence information use, management and technology adoption; and age captures life-cycle and experience-related differences that may shape production decisions and accumulated farming knowledge. Credit, extension, input access, land rights, household bargaining and wider institutional norms remain important structural mechanisms, but they are not treated as econometrically identified determinants of yield in this study. Although gender differences in agricultural productivity are well documented in Nigeria and sub-Saharan Africa, three gaps motivate the present study. Oseni et al. (2015) used nationally derived agricultural data and Oaxaca-Blinder decomposition to examine gender productivity differentials, while Olakojo (2017) assessed gender productivity gaps across several major crops. These studies establish broader differences in productivity and resource endowments but do not specifically compare gender-associated yield differentials in cowpea and soybean. Crop-specific studies provide additional evidence: Kamara et al. (2022), for example, examined gender differences in the yield and revenue effects of improved soybean adoption in northern Nigeria, while related work has documented gender inequalities in technology adoption, resource access and market participation (Daudu et al., 2025; Josephson, 2025; Maereka et al., 2023; Msosa, 2022). However, evidence remains limited regarding how gendered resource control and agricultural decision-making characterize the production contexts of cowpea and soybean, and whether yield differences persist among farmers with comparable observed characteristics. The present study therefore addresses crop-specific, geographical and analytical gaps without claiming to identify the causal effects of the full range of structural and institutional barriers. It focuses on three research questions: (1) How do male- and female-headed farming households differ in the observed dimensions of productive-resource access and agricultural decision-making captured by the survey? (2) Does a gender-associated yield differential persist after accounting for farm size, age and education? (3) To what extent is the observed yield differential associated with differences in these observable endowments and their estimated returns? The study makes a valuable contribution by connecting descriptive evidence on gendered production conditions with a deliberately bounded econometric assessment of yield differentials. PSM compares yields among male- and female-headed farms with similar observed characteristics; Oaxaca-Blinder decomposition separates the mean yield differential into components associated with observed endowments, estimated returns and their interaction; and an OLS gender × crop interaction model assesses whether the gender-associated yield difference varies between cowpea and soybean. In this way, the analysis moves beyond documenting women’s visibility while remaining explicit about its empirical boundaries: it estimates gender-associated productivity differences and their observable correlates rather than the causal effects of wider institutional and social mechanisms.

## 2 Materials and methods

### 2.1 Study area

Nigeria was selected as the study setting because of its importance in African legume production and as a major market for legume grain and fodder (Omoigui et al., 2020). The study focused on major cowpea- and soybean-producing areas, particularly within the Guinea and Sudan Savanna agroecological zones where these crops are widely cultivated. Six states: Benue, Jigawa, Kaduna, Kano, Nasarawa, and Niger (Figure 1) were purposively selected based on their importance in the production of the target legumes and their relevance to the farming systems examined. The selected states capture variation across important legume-producing contexts and production environments. However, because state selection was purposive rather than probability-based, the geographical coverage was not designed to produce estimates that are statistically representative of Nigeria’s national farming population. Findings are therefore interpreted as evidence from the surveyed legume-producing contexts rather than national population estimates.

**Figure 1:**
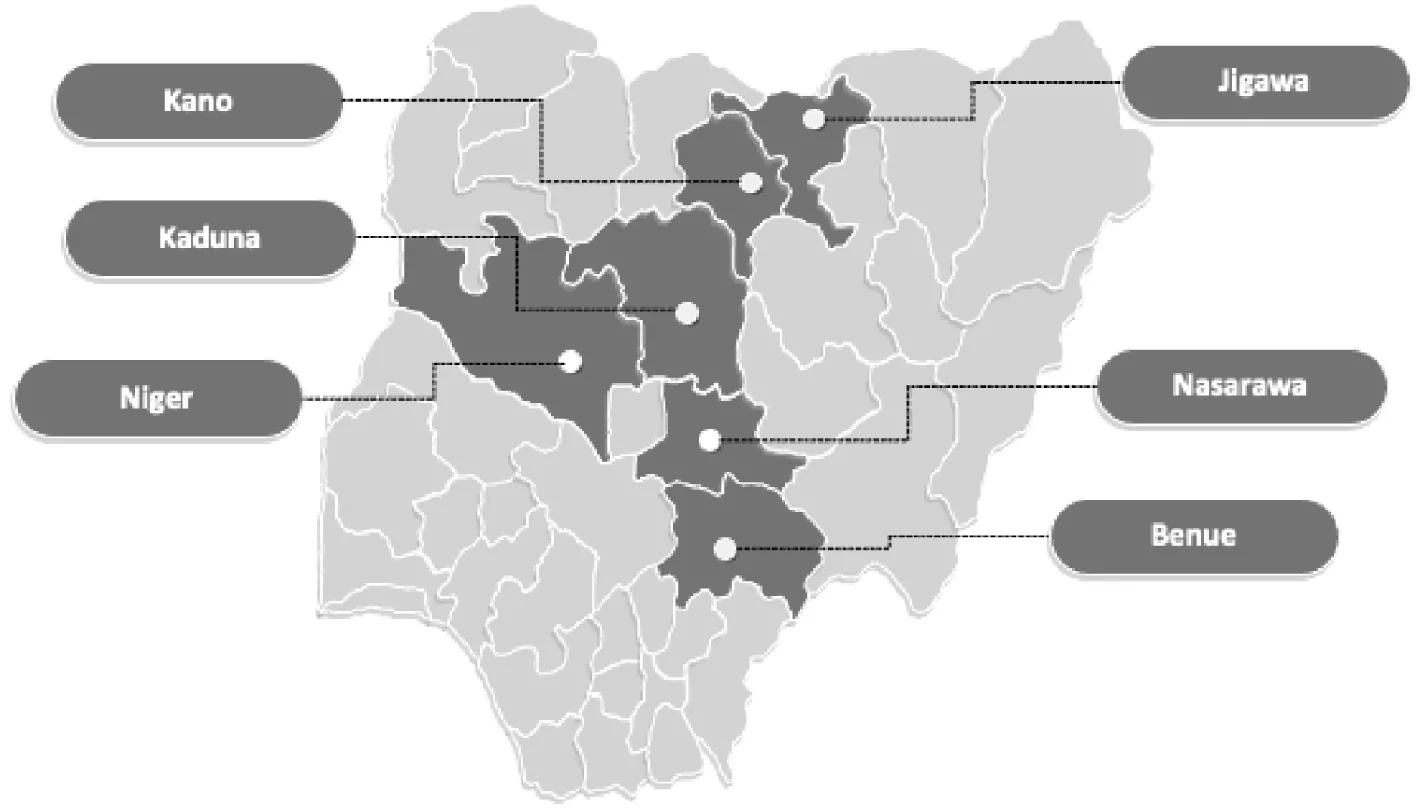
Map of the study area. YourFreeTemplates.com (2016). Free Nigeria Map with 36 States. Creative Commons Attribution–NoDerivatives 4.0 International (CC BY-ND 4.0). Source: https://yourfreetemplates.com/free-nigeria-editable-map/.

### 2.2 Study population

The broader survey population comprised actors involved in cowpea and soybean production and seed systems in the selected states, including farmers, seed companies, researchers, and representatives of supporting organizations. For the present gender-productivity analysis, however, the analytical population was restricted to farmer respondents engaged in cowpea and/or soybean production for whom the relevant farm-level production and socioeconomic information was available. Institutional actors were treated as a separate respondent category and excluded from the farmer-level econometric analysis. The farmer or farm household was therefore the principal unit of analysis for assessing gender differences in resource access, agricultural decision-making, and productivity.

### 2.3 Sampling strategy

A purposive sampling approach was used to recruit respondents actively involved in cowpea and/or soybean production and related seed system activities across the six selected states. The study was designed to examine gender differences among active legume producers rather than to generate nationally representative prevalence estimates. Selection therefore targeted respondents with direct and relevant experience in cowpea and soybean production and related seed system activities, resulting in an overall survey sample of 1,010 respondents (Table 1). Both male- and female-headed farms were eligible for participation, and gender was not used as a pre-selection criterion. The sample was not proportionally allocated according to gender, state population, or Nigeria’s national farming population. Consequently, the observed gender distribution reflects the respondents reached through the purposive recruitment process rather than the gender composition of Nigeria’s farming population. Because the sampling design was non-random, population-level generalization is approached with caution. PSM was subsequently employed to improve comparability between male- and female-headed farms with respect to selected observed characteristics. Importantly, PSM does not correct the non-probability sampling design or eliminate bias arising from unobserved characteristics.

**Table 1:** Distribution of survey respondents by state.

States	Benue	Jigawa	Kaduna	Kano	Nasarawa	Niger	All
LGAs	Buruku (25)	Birnin Kudu (20)	Ikara (24)	Awazara (4)	Obi (78)	Gbako (36)	
	Gboko (73)	Dutse (20)	Kauru (66)	Bichi (40)		Katcha (15)	
	Otukpo (1)	Gwaram (16)	Kubau (11)	Dalili (1)		Lavun (36)	
	Tarka (31)	Kila (3)	Sabon Gari (58)	Dawakin Kudu (1)			
	Ushongo (22)		Soba (60)	Gaya (35)			
	Gwer West (6)		Makarfi (6)	Gwarzo (62)			
	Makurdi (8)		Kaduna (2)	Kiru (39)			
				Kura (39)			
				Nassarawa (6)			
				Sumaila (63)			
				Takai (61)			
				Tarauni (1)			
				Tofa (40)			
				Kano (1)			
Total	166	59	227	393	78	87	1,010
# Men	118	57	168	381	40	81	845
# Women	48	2	59	12	38	6	165

Figures in parentheses are the sample size.

### 2.4 Data collection process

The study employed cross-sectional survey data collected between October and December 2022. Trained enumerators administered a semi-structured questionnaire in English, with local-language interpretation where required, through face-to-face interviews and KoboCollect. The questionnaire captured household and farmer characteristics, farm size, production experience, resource access, agricultural decision-making, production practices, and crop yields. Open-ended responses were used to clarify respondents’ circumstances and support contextual interpretation but were not analyzed as a separate qualitative dataset. Written informed consent was obtained before participation. The dataset was checked for completeness, internal consistency, and outliers before analysis in Stata.

Among the 1,010 respondents, 845 were male and 165 were female. The sample was not proportionally allocated by gender, state population, or national farmer population, and no gender quota was imposed during recruitment. The observed gender distribution, therefore, reflects the respondents reached through the study’s purposive recruitment process rather than the gender composition of Nigeria’s farming population. Crop-specific participation was highly unbalanced by gender: 320 respondents reported growing cowpea (315 male and 5 female), and 407 reported growing soybean (375 male and 32 female) (Table 2).

**Table 2:** Farmers growing cowpea and soybean.

**Crop**	**Female**	**Male**	**Total**
Cowpea	5	315	320
Soybean	32	375	407

The study is therefore characterized primarily as a cross-sectional quantitative survey. Descriptive responses on resource access and decision-making are used to characterize the structural gender context, whereas the inferential yield-gap analysis relies on the quantitative farmer-level observations available for the PSM, Oaxaca-Blinder, and OLS specifications.

### 2.5 Data analysis

The analysis combined descriptive and econometric approaches. Descriptive statistics were used to summarize respondent and enterprise characteristics and to characterize gender differences in access to productive resources and in agricultural decision-making, including land ownership and control, access to inputs and services, production decisions, commercialization, and control over income. Independent-samples *t*-tests and chi-square tests were used, where appropriate, to assess gender differences in continuous and categorical variables. These indicators provide sociological evidence on the gendered conditions under which legume production occurs; because they are not included in the principal yield models, they are not interpreted as econometrically identified or causal determinants of yield.

On the other hand, the econometric analysis had a more focused goal: to estimate yield differences by gender, based on a streamlined set of observable factors that are both theoretically significant and consistently measured across the sample. Farm size is a key indicator of access to essential productive assets and of production scale. Years of formal education reflect the human capital necessary for information processing, management, and technology use, while age accounts for life-cycle and experience-related variations that could affect farm decisions and accumulated knowledge. Thus, these three variables were the main covariates used in the PSM and Oaxaca-Blinder models. Broader indicators including access to fertilizer, improved seed, credit, and extension services, as well as land rights and decision-making measures were not incorporated because comparable and sufficiently complete observations were not consistently available in the econometric analytical sample. Their exclusion means the models do not capture the full set of structural and institutional mechanisms that may be associated with gender differences in productivity. The resulting estimates are therefore interpreted as conditional on the included observable endowments, not as a comprehensive causal explanation of the gender yield gap. The econometric analysis proceeded in three stages: (i) the Propensity Score Matching (PSM) was used to compare yields between male- and female-headed farms with similar observed characteristics (Lachaud, 2024); (ii) the Oaxaca-Blinder decomposition was used to partition the mean yield differential into endowment, coefficient, and interaction components; (iii) an OLS model incorporating a gender-by-crop interaction was estimated to assess whether the gender-yield relationship differed between cowpea and soybean. All analyses were conducted in Stata.

### 2.6 Econometric techniques

#### 2.6.1 Propensity score matching

Propensity Score Matching (PSM) was used as a balancing technique to estimate the conditional yield differential between male- and female-headed farms with similar observed characteristics. Because the sex of the household head is not a manipulable treatment, the Average Treatment Effect on the Treated (ATT) is interpreted as a matched conditional association rather than a causal effect of gender. The propensity-score model included farm size, age of the household head, and years of formal education because these variables are theoretically relevant to farm productivity and were consistently observed across the analytical sample.

In the first stage, the probability of belonging to the male-headed group was estimated using a probit specification based on the observed covariates, as expressed in Equation 1:


$$P(X_{i})=\mathrm{Pr}(\mathrm{se}x_{i}=1|X_{i})=F(\alpha +X_{i}^{'}\beta )$$ (1)

Where:

F(⋅) is the cumulative normal distribution, and.

$X_{i}$ represents a vector of observable characteristics such as farm size (ha), age of household head (years), and education (years of formal schooling).

In the second stage, male- and female-headed farms were matched using the nearest-neighbor algorithm under the common support condition. Covariate balance was assessed using standardized mean differences and variance ratios. The ATT was then computed as expressed in Equation 2:


$${\hat{\mathrm{ATT}}}_{\mathrm{PSM}}=\frac{1}{N_{T}}\sum_{i\in T}(Y_{i}-\sum_{j\in C(i)}w_{\mathrm{ij}}Y_{j})$$ (2)

Where:

$Y_{i}$ denotes the yield measured in kilograms per hectare (kg/ha),

$N_{T}$ is the number of observations in the focal (male-headed) group, and.

$w_{\mathrm{ij}}$ are matching weights for control observations.

The validity of the matched estimate depends on sufficient overlap in propensity scores and adequate balance in observed covariates between male- and female-headed farms. These conditions should therefore be considered when interpreting the PSM results. Given the observational design, matching can account only for the observed characteristics included in the propensity-score model and cannot eliminate differences arising from unobserved factors. Accordingly, the estimated ATT is interpreted as a conditional yield difference between matched male- and female-headed farms rather than as a causal effect of gender (Hashimoto and Yasunaga, 2022). Residual confounding from unobserved characteristics may remain.

#### 2.6.2 Oaxaca-Blinder decomposition

The Oaxaca-Blinder decomposition was used to examine the sources of the mean yield differential between male- and female-headed farms (Blinder, 1973; Oaxaca, 1973). Gender defines the two comparison groups (male = 1; female = 0), while crop yield is measured in kg/ha. For clarity, the reported differential expressed as shown in Equation 3 is defined as the female mean yield minus the male mean yield; a negative value, therefore, indicates a male yield advantage.


$$\varphi =E(Y_{1})-E(Y_{0})$$ (3)

The outcome model can be modeled as presented in Equation 4:


$$Y_{\tau }=X_{\tau }^{'}\beta_{\tau }\varepsilon \tau ,E(\varepsilon \tau =0\;\;\tau \varepsilon (1,0))$$ (4)

Where:

𝑌𝜏 is the yield for group *τ*, 𝑋′𝜏 are observed characteristics included in the decomposition: farm size (ha), age (years), and education (years of formal schooling). Gender itself is not entered as a covariate because it defines the two groups being decomposed.

𝛽𝜏 are the parameters to be estimated, 𝜖𝜏 is the error term, which is normally distributed with zero mean and constant variance.

Therefore, Equation 5 is derived by substituting Equation 4 into Equation 3 as follows:


$$\varphi =E(Y_{1})-E(Y_{0})=E(X_{1}^{'})-E(X_{0}^{'})\beta_{0}$$ (5)

To compute the difference in the outcome variable (𝜑) that is due to differences in the amounts of observed variables between the two groups of gender (male and female), Equation 5 can be rearranged to give a three-fold decomposition framework as follows:

The Oaxaca–Blinder decomposition expresses the mean yield differential between male- and female-headed farms in terms of their observed characteristics and estimated coefficients, as shown in Equation 6:


$$\begin{array}{l} \beta ={\left\{E(X_{1})-E(X_{0})\right\}}^{'}\beta_{0}+E{\left(X_{0}\right)}^{'}(\beta_{1}-\beta_{0})+\\ {\left\{E(X_{1})-E(X_{0})\right\}}^{'}(\beta_{1}-\beta_{0}) \end{array}$$ (6)

This can be further decomposed into three additive components: the portion of the gap explained by differences in endowments/resources (E), differences in returns to those resources or coefficients (C), and the interaction between endowments and coefficients (I) (Oaxaca and Ransom, 1994). Mathematically, the overall yield differential was further decomposed into endowment, coefficient, and interaction components, as presented in Equation 7:


φ=E+C+1 (7)

Where:

𝐸 (resource amount or endowment effect) = {𝐸(𝑋1)−𝐸(𝑋0)}′𝛽0,

𝐶 (returns or efficiency or coefficient effect) = (𝑋0)′(𝛽1− 𝛽0),

𝐼 (interaction term) = {(𝑋1)−𝐸(𝑋0)}′(𝛽1− 𝛽0).

The decomposition is reported using female-headed farms as the reference group. Accordingly, the sign and magnitude of each component should be interpreted relative to the stated female-minus-male differential. The endowment component indicates the contribution of group differences in the observed covariates; the coefficient component captures differences in estimated returns to those covariates; and the interaction term captures their joint contribution (Fortin et al., 2011). Conversely, the model could also be reformulated from the male perspective, although the interpretations would remain analogous. Oaxaca-Blinder decomposition is used here as a group-comparison framework rather than as a treatment-effect estimator. It does not estimate technical efficiency and should not be interpreted as demonstrating inherent productivity differences between men and women (Rahimi and Hashemi Nazari, 2021).

Farm size was measured in hectares, age in years, education in years of formal schooling, and yield in kg/ha. Crop yield was calculated from respondents’ self-reported production and cultivated area. Because both measures were self-reported, yield may be affected by recall and measurement error and is therefore treated as a survey-based measure of farm productivity rather than an independently verified plot-level measure. Resource-access and decision-making indicators were analyzed descriptively because comparable and sufficiently complete observations were not available for their consistent inclusion in the econometric analytical sample; consequently, the decomposition is limited to the observed endowments included in the model.

To assess whether the gender-yield association differed between cowpea and soybean, an OLS model with a gender-crop interaction term was estimated before pooling the crops for subsequent gender-gap analyses. The interaction specification provides a direct test of whether the gender coefficient varies by crop. Heteroskedasticity-robust standard errors were used because cross-sectional farm data commonly exhibit non-constant error variance. The model was specified as:

Yᵢ = β₀ + β₁Maleᵢ + β₂Cropᵢ + β₃(Maleᵢ × Cropᵢ) + β₄Ageᵢ + β₅Educationᵢ + β₆FarmSizeᵢ + εᵢ

Where:

Male is coded 1 for male-headed farms and 0 for female-headed farms.

Crop is coded 0 = cowpea and 1 = soybean, making cowpea the reference crop.

Accordingly, β₁ represents the conditional gender-associated yield difference for cowpea,

β₂ represents the soybean-cowpea yield difference for the reference gender group, and

β₃ tests whether the gender-associated yield differential differs between soybean and cowpea.

Failure to reject H₀: β₃ = 0 provides statistical support for pooling with respect to the gender-yield relationship, but does not imply that the two crops are equivalent in their agronomic, market or gender characteristics.

Table 3 summarises the key study variables, their definitions, and their analytical roles, distinguishing variables included in the econometric models from resource-access and decision-making indicators examined descriptively.

**Table 3:** Key variables and analytical role.

Variable	Definition	Analytical use
Farm size	Total farm area owned/cultivated by farmer (ha)	PSM, Oaxaca-Blinder, OLS
Education	Years of formal education	PSM, Oaxaca-Blinder, OLS
Age	Age of farmer/household head (years)	PSM, Oaxaca-Blinder, OLS
Resource access	Indicators of access to farming inputs and services	Descriptive analysis
Decision-making	Responsibility for key land, production, seed, sale and income decisions	Descriptive analysis

## 3 Results

### 3.1 Gendered resource control and decision-making

Table 4 reveals a consistent gender imbalance in land ownership, resource control, production decisions and commercialisation, with men dominating each dimension. Male respondents account for about 85% of sole land ownership, compared with about 9% for women and 7% under joint ownership. Similar patterns occur in decisions to rent, borrow, or share land; control land use; choose crops and seed varieties; sell produce; and determine how to use sales income. The statistical tests (*p* < 0.001 across the reported dimensions) indicate marked gender differences in these distributions. These patterns are consistent with the broader structural context described in the Introduction: women may participate visibly in legume production while having less control over productive assets and key decisions (Bhati and Arya, 2025; Celik and Yildiz, 2024; Atreya et al., 2022; Olakojo, 2017). However, because land ownership, leasehold rights, income control, and other decision-making indicators were not included in the econometric yield models due to incomplete comparable observations, Table 4 is interpreted as complementary sociological evidence rather than as evidence of their direct effect on yield. The descriptive and econometric analyses therefore answer different but related questions: the former documents unequal conditions of participation and control, while the latter estimates gender-associated yield differences conditional on farm size, age and education. No causal pathway is claimed from the inequalities in Table 4 to the estimated yield gap.

**Table 4:** Gendered land ownership and agricultural decision-making in Nigeria’s legume systems.

**Dimension**	**Decision-maker/ Owner**	**Percentage**	**χ**^**2**^ **Statistic**
Land ownership	Man	84.5%	412.91*
	Woman	8.6%	
	Jointly	6.9%	
Decision to rent/ borrow/ share land	Man	80.7%	303.26*
	Woman	8.7%	
	Jointly	10.6%	
Control over use of land	Man	84.5%	348.13*
	Woman	6.7%	
	Jointly	8.7%	
Decision on what to grow on the land	Man	79.4%	373.44*
	Woman	6.9%	
	Jointly	13.7%	
Decision about seed variety to plant	Man	78.7%	386.27*
	Woman	8.1%	
	Jointly	13.2%	
Decision to sell	Man	78.8%	109.28*
	Woman	4.4%	
	Jointly	16.8%	
Sale of seeds	Man	83.8%	93.00*
	Woman	6.5%	
	Jointly	9.7%	
Decision about use of income earned from seed sale	Man	71.9%	168.92*
	Woman	3.0%	
	Jointly	25.2%	

* indicates statistical significance at *p* < 0.10.

### 3.2 Farmer involvement in cowpea and soybean production

Table 5 summarizes farmer involvement in soybean and cowpea enterprises. The results show that male-headed farms have an average of about 15 years of grain-production experience, while female-headed farms have an average of only 9 years. This difference in experience is statistically significant (*p* < 0.01) and unfavorable for women. Additionally, the average land area dedicated to legume production is 1.3 hectares for male-headed farms compared to 0.7 hectares for female-headed farms, a significant difference at the 10% level. These differences identify observable endowments that may contribute to gender-associated yield disparities. Other enterprise indicators, including several measures of seed and grain quantities, did not show statistically significant gender differences in the available sample. The observed differences in production experience and farm size are broadly consistent with previous studies that report gender disparities in access to productive resources (Kamara et al., 2025; Jaleta et al., 2023).

**Table 5:** Gender analysis of household involvement in legume production enterprises.

Legume enterprise characteristics	All	Male	Female	*t*-test
Grain production experience (yrs)	14.4 (626)	14.8 (591)	8.5 (35)	−3.5***
Seed production experience (yrs)	7.1 (391)	7.2 (378)	5.1 (13)	−1.0
Variety planting experience (yrs)	16.3 (643)	15.4 (616)	36.4 (27)	0.8
Farm size (ha)	1.3 (639)	1.3 (611)	0.7 (28)	−1.7*
Seed quantity (kg/ha)	46.2 (644)	45.4 (619)	65.9 (25)	1.0
Seed cost per kg (₦)	915.6 (607)	933.7 (585)	436.6 (22)	−0.6
Yield (kg/ha)	1037.0 (549)	1051.3 (523)	750.0 (26)	−0.8
Quantity saved as seed (kg)	702.0 (276)	707.0 (272)	356.3 (4)	−0.5
Duration of seed storage (month)	14.7 (295)	14.8 (291)	7.5 (4)	−0.2
Quantity of seed sold (kg)	999.2 (235)	1011.6 (232)	35.0 (3)	−0.1
Distance to main seed buyer (km)	9.3 (258)	9.3 (254)	8.3 (4)	−0.1
Price of seed sold per kg (₦)	811.3 (252)	814.3 (249)	560.0 (3)	−1.1
Seed sold to main buyer qty. (kg)	745.6 (244)	754.6 (241)	18.3 (3)	−0.8
Quantity of seed sold as grain (kg)	844.0 (63)	861.9 (61)	300.0 (2)	−0.3
Quantity of grain harvested (kg)	809.2 (567)	817.2 (536)	671.5 (31)	−0.9
Quantity saved as grain (kg)	570.4 (567)	578.5 (536)	429.6 (31)	−1.0
Duration of grain storage (month)	12.7 (565)	13.2 (534)	5.0 (31)	−0.5
Quantity of grain sold (kg)	1443.5 (564)	1493.8 (533)	577.2 (31)	−0.3
Distance to main grain buyer (km)	5.4 (551)	5.5 (522)	4.5 (29)	−0.4
Price of grain sold per kg (₦)	742.6 (531)	749.0 (504)	623.0 (27)	−1.2
Average quantity of grain purchased by main buyers qty. (Kg)	1426.1 (512)	1474.2 (486)	527.0 (26)	−0.3

Figures in parentheses represent the frequency. * indicates significance at *p* < 0.10; *** indicates significance at *p* < 0.01.

#### 3.2.1 Farm Enterprise characteristics by gender

Table 6 presents gender differences in selected legume enterprise characteristics. The distribution of farmers across production-purpose categories differed significantly by gender (χ^2^ = 13.9, *p* < 0.01), as did the reported reason for planting legumes (χ^2^ = 12.6, *p* < 0.01). Seed classification also differed at the 10% significance level (χ^2^ = 3.6, *p* < 0.10), with improved seed more frequently reported among male farmers. These descriptive findings reinforce the broader pattern of unequal participation and access but are not interpreted as causal drivers of yield. Across both gender groups, about 59% of farmers reported expanding their cultivated land over the preceding four seasons, 12% reported a decrease, and 29% reported no change (Figure 2).

**Table 6:** Gendered legume enterprise characteristics.

Legume enterprise characteristics	All %	Male %	Female %	Chi^2^
Proportion planting legumes				
Seed	100 (122)	98.3 (120)	1.6 (2)	
Grain	100 (414)	92.3 (382)	7.7 (32)	
Both	100 (191)	27.3 (188)	1.6 (3)	
All	100 (727)	94.9 (690)	5.1 (37)	13.9***
Reasons for planting legumes				
Seed	100 (158)	98.1 (155)	1.9 (3)	
Grain	100 (330)	92.7 (306)	7.3 (24)	
Both	100 (164)	98.8 (162)	1.2 (2)	
All	100 (652)	95.6 (623)	4.5 (29)	12.6***
Seed classification				
Local	100 (182)	98.4 (179)	1.7 (3)	
Improved	100 (448)	95.1 (426)	4.9 (22)	
All	100 (630)	96.0 (605)	4.0 (25)	3.6*
Main Buyer of the seed				
Farmer group	100 (42)	100.0 (42)	0.0 (0)	
Fellow farmer	100 (156)	90.1 (153)	1.9 (3)	
NGO	100 (1)	100.0 (1)	0.0 (0)	
Project	100 (19)	100.0 (19)	0.0 (0)	
Seed Company	100 (8)	100.0 (8)	0.0 (0)	
Other	100 (29)	100.0 (29)	0.0 (0)	
All	100 (255)	98.8 (1)	1.2 (3)	1.9
Sell harvested seed				
No	100 (76)	100.0 (76)	0.0 (0)	
Yes	100 (240)	98.3 (236)	1.7 (4)	
All	100 (316)	98.7 (312)	1.3 (4)	1.3
Seed bank availability				
No	100 (480)	96.5 (463)	3.5 (17)	
Yes	100 (30)	96.7 (29)	3.3 (1)	
All	100 (510)	96.5 (492)	3.5 (18)	0.0

Values in parentheses represent the number of stakeholders. * indicates significance at *p* < 0.10; *** indicates significance at *p* < 0.01.

**Figure 2:**
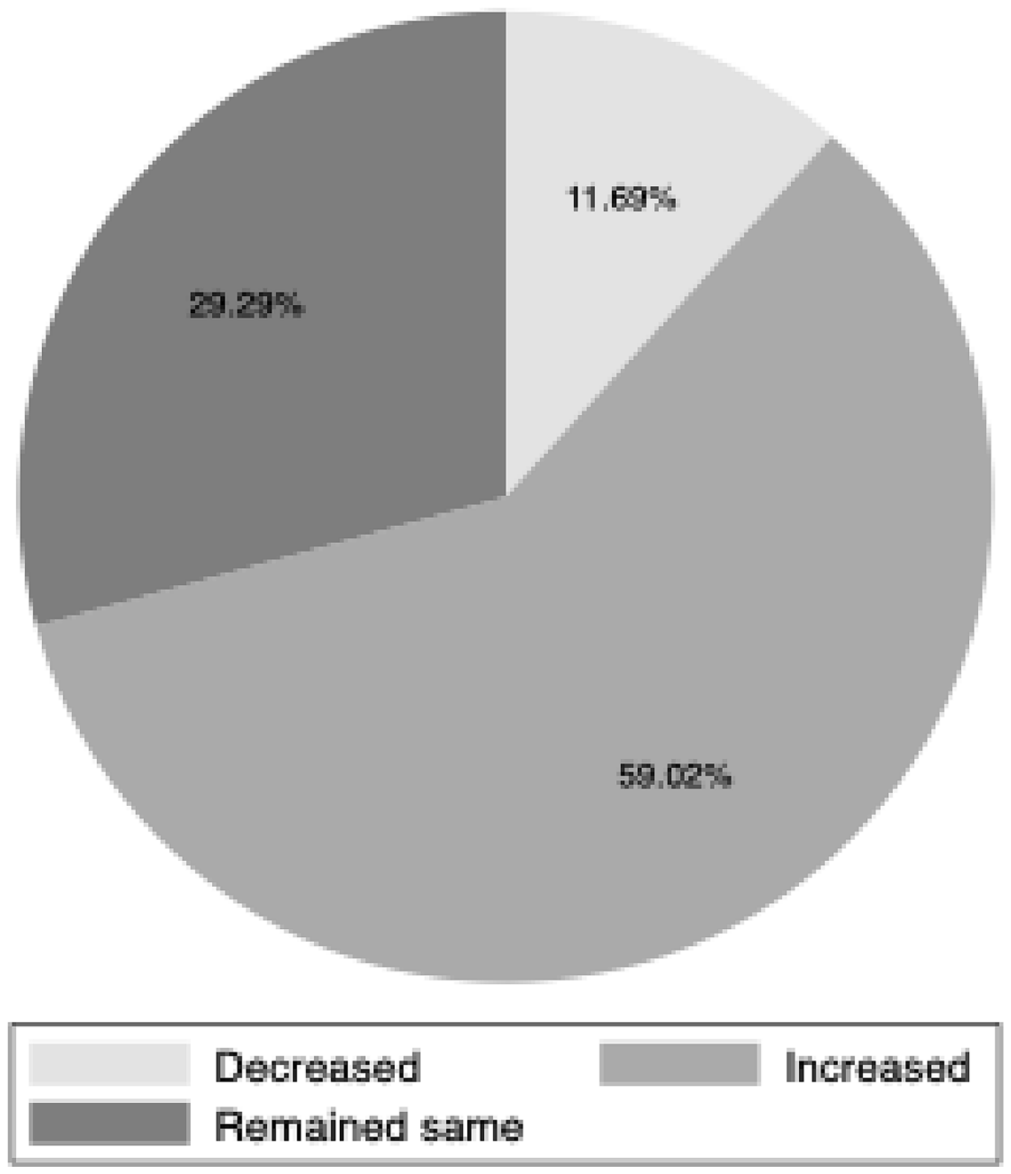
Change in cultivated area for cowpea and soybean farms over the previous four seasons.

### 3.3 Gender-associated yield differentials

#### 3.3.1 Propensity score matching

PSM was used to compare yields between male- and female-headed farms after balancing the groups on farm size, age, and education (Table 7). The matched male-headed group recorded a mean yield of 1,070.8 kg/ha compared with 641.5 kg/ha for the matched comparison group, producing a matched ATT estimate of 429.3 kg/ha (SE = 253.01; *t* = 1.70; *p* < 0.10). This estimate is interpreted as a conditional gender-associated yield difference among farms comparable on the observed matching variables, rather than as a causal effect of gender. Post-matching standardized mean differences for the included covariates were below 10%, and the reported variance ratios fell within commonly used balance ranges, indicating improved observed covariate balance. The result is broadly consistent with previous studies reporting male advantages in observed agricultural productivity (Aguilar et al., 2015; Muricho et al., 2020).

**Table 7:** Propensity score matching results.

Variable	Coefficient	Standard error	*z*-test
Farm size	0.4957	0.1895	2.62***
Age of household head	−0.0002	0.0003	0.90
Education	0.0006	0.0003	1.89*
Constant	1.0414	0.1768	5.89***

**Yield comparison**	**Treated**	**Controls**	**Difference**	**Standard error**	***t*-stat**
Yield (unmatched kg/ha)	1,070.800	730.435	340.366	402.522	0.85
ATT	1,070.800	641.457	429.343	253.014	1.70*

* indicates significance at *p* < 0.10; *** indicates significance at *p* < 0.01.

#### 3.3.2 Oaxaca-Blinder decomposition

The Oaxaca-Blinder decomposition (Table 8) provides a complementary interpretation of the gender yield differential. With the differential defined as female minus male, predicted mean yield was 730.44 kg/ha for female-headed farms and 1,070.80 kg/ha for male-headed farms, producing a statistically significant differential of −340.37 kg/ha (*z* = −2.22; *p* < 0.05). The negative sign indicates a lower predicted mean yield for female-headed farms in the analytical sample; it does not indicate technical efficiency or an inherent productivity difference. The endowment component was −438.91 kg/ha and statistically significant (*z* = −5.42; *p* < 0.01), indicating that differences in the observed characteristics inclu ded in the model farm size, age, and education substantially contribute to the estimated yield gap. By contrast, the coefficient component (−1,506.08 kg/ha; *z* = −0.58) and interaction component (1,604.63 kg/ha; *z* = 0.62) were not statistically significant. Their large point estimates are accompanied by substantial estimation uncertainty and should not be interpreted as evidence of different technical efficiency or systematically different returns to resources. Thus, the PSM and Oaxaca-Blinder results are complementary rather than contradictory: the PSM identifies a conditional male yield advantage after balancing on observed characteristics, while the decomposition shows that observed endowment differences contribute significantly to the mean female–male yield gap. Neither method establishes that women are technically more or less efficient.

**Table 8:** Oaxaca-Blinder decomposition of the gender yield differential.

Component	Estimate	Standard error	*z*-statistic
Female predicted yield	730.435	118.745	6.15***
Male predicted yield	1,070.801	96.759	11.07***
Female–male difference	−340.366	153.176	−2.22**
Endowments	−438.909	80.955	−5.42***
Coefficients	−1,506.083	2,588.084	−0.58
Interaction	1,604.626	2,584.853	0.62

** indicates significance at *p* < 0.05; *** indicates significance at *p* < 0.01.

These endowment effects should not be interpreted as capturing the broader inequalities in land control, decision-making, input access, or institutional conditions documented descriptively, because these dimensions were not included in the decomposition model.

#### 3.3.3 Crop heterogeneity and rationale for pooled analysis

Given the very small female cowpea subsample (*n* = 5), separate crop-specific gender-gap models would yield imprecise and potentially unstable estimates. The principal econometric analysis therefore pooled cowpea and soybean observations. Because the two crops have distinct agronomic characteristics, market structures and potentially different gender relations, pooling was assessed rather than assumed. An OLS model incorporating a gender × crop interaction was estimated to test whether the gender-associated yield differential differed significantly between cowpea and soybean (Table 9). With cowpea as the reference crop (0 = cowpea; 1 = soybean), the interaction coefficient was −236.55 and was not statistically significant (*t* = −1.43; *p* = 0.152). The available sample therefore provides insufficient evidence that the gender-yield association differs between the two crops. This supports pooling for the limited purpose of estimating an overall gender-associated yield differential, but it does not establish equivalence between the crops. In particular, the very small number of female cowpea growers substantially limits statistical power to detect crop-specific heterogeneity. The model explains approximately 18% of observed yield variation (*R*^2^ = 0.1815), and heteroskedasticity-robust standard errors were used to maintain valid inference under possible non-constant error variance.

**Table 9:** OLS estimates and test of crop-specific gender effects.

Variable	Coefficient	Robust standard error	*t*-statistic	P > |t|
Gender	247.3934	114.3295	2.16**	0.031
Crop	−236.547	145.0483	−1.63	0.105
Gender × Crop	−236.547	164.9013	−1.43	0.152
Age	−0.1615	0.0753	−2.15**	0.032
Education	0.2307	0.0500	4.61***	0.000
Farm size	374.0388	145.0840	2.58**	0.010
Constant	−159.7923	155.4980	−1.03	0.305
Observations	421			
*R* ^2^	0.1815			
Overall F-statistic	37.53			

Gender × crop test: *F*(1,414) = 2.06, *p* = 0.1522. Crop is coded 0 = cowpea and 1 = soybean; therefore, cowpea is the reference crop. The gender × crop interaction tests whether the gender-associated yield differential differs between cowpea and soybean. Robust standard errors are reported. ***p < 0.01; **p < 0.05; *p < 0.10.

### 3.4 Study limitations and future research

Several limitations affect the interpretation of the findings. First, purposive sampling limits national generalization. Second, the gender imbalance in the analytical sample, particularly the five female cowpea growers, reduces the precision of gender comparisons, may increase sampling variability and standard errors, and makes the estimates more sensitive to individual observations. It also precludes reliable crop-specific gender-gap models and limits the statistical power to detect differences in the gender–yield relationship between cowpea and soybean. The pooled estimates should therefore be interpreted with caution and not considered representative of crop-specific gender effects. Third, the cross-sectional design and the restricted set of covariates limit causal interpretation. The PSM accounts only for the observed characteristics included in the model: farm size, age, and education and cannot account for unobserved differences between male- and female-headed farms. If omitted factors such as input use, credit and extension access, management practices, land quality, land rights, or decision-making authority are associated with both gender and yield, the estimated ATT may be biased. Similarly, in the Oaxaca-Blinder decomposition, omitted factors may influence the estimated endowment, coefficient, or interaction components. The significant endowment component may therefore over- or underestimate the contribution of the observed characteristics and should be interpreted as conditional on the variables included in the model rather than as a complete causal explanation of the gender yield gap.

Finally, self-reported production and cultivated area may contain measurement error. Measurement error in farm size may attenuate its estimated association with yield, while errors in cultivated area may also affect the calculated yield because area enters its denominator. The overall direction of these effects is therefore uncertain. Future research should employ larger, more gender-balanced and crop-specific samples; probability-based sampling where national inference is intended; objective or GPS-based land-area measurement; and longitudinal data incorporating richer measures of land rights, input and seed access, extension, finance, management practices, intra-household decision-making, land quality, and agroecological conditions.

## 4 Conclusion

This study identifies gender-associated differences in cowpea and soybean farming within the surveyed legume-producing areas of Northern Nigeria. PSM indicates a conditional male yield advantage among farms matched on farm size, age and education, while the Oaxaca-Blinder decomposition identifies a significant female–male yield differential and shows that differences in the observed endowments included in the model contribute substantially to that gap. The non-significant coefficient and interaction components provide no evidence of a female technical-efficiency advantage or systematically different returns to the observed characteristics. These results do not establish that inequalities in land rights, seed access, credit, extension, or decision-making cause the estimated yield differential, because those mechanisms were not included in the principal econometric models.

Moving beyond women’s visibility in legume production therefore requires distinguishing between interventions directly supported by the econometric evidence and wider structural constraints identified descriptively. The present results support attention to observable productive endowments, while the descriptive findings point to institutional and intra-household domains requiring deeper investigation. Future research incorporating land rights, seed and input access, extension, finance, management practices and intra-household decision-making into richer longitudinal or purpose-designed models would help establish which mechanisms most effectively narrow gender-associated productivity gaps and strengthen women’s substantive empowerment in legume production.

## Data Availability

The original contributions presented in the study are included in the article/supplementary material, further inquiries can be directed to the corresponding author/s.
